# Evaluation of nasal mucociliary clearance by saccharine test in rheumatoid arthritis

**DOI:** 10.1016/j.bjorl.2021.08.005

**Published:** 2021-10-17

**Authors:** Hilal Yücel, Serpil Ergülü Eşmen

**Affiliations:** a University of Health Sciences, Department of Otorhinolaryngology Head and Neck Surgery, Konya City Hospital, Konya, Turkey; b University of Health Sciences, Department of Rheumatology, Konya City Hospital, Konya, Turkey

**Keywords:** Rheumatoid arthritis, Nasal mucociliary clearance, Disease activity score

## Abstract

**Objective:**

We aimed to reveal whether there is nasal involvement by examining the Nasal Mucociliary Clearance (NMC) and the relationship between this activity and disease severity in Rheumatoid Arthritis (RA) patients.

**Methods:**

In this prospective study, NMC time, disease activity (Disease Activity Score 28) and blood parameters of RA patients (n = 87) were investigated and compared with the healthy control group (n = 50). In addition, the relationship between DAS 28 and NMC was investigated.

**Results:**

The mean NMC of the RA group was 9.51 ± 3.54 min, the mean NMC of the control group was 8.69 ± 2.85 min, and there was no significant difference between the two groups. There was no correlation between NMC and disease duration, and DAS 28. The mean NMC of the RA patients with Anti Cyclic Citrulled Peptide (Anti-CCP) positive was significantly higher than the negative ones.

**Conclusion:**

Although there was no significant difference in NMC values between the RA and control group, the NMC of the Anti-CCP positive patients was higher.

**Level of evidence:**

Level 2.

## Highlights

• Nasal involvement has not been fully addressed in Rheumatoid Arthritis (RA).
• Nasal Mucociliary Clearance (NMC) time of RA patients was within normal limits and was similar to the control group.
• Anti-cyclic citrated peptide (Anti-CCP) positivity may affect NMC time in RA patients.

## Introduction

Rheumatoid Arthritis (RA) is a chronic, autoimmune, and systemic disease that occurs as an inflammation of the synovial joints and causes joint destruction and deformity. It is estimated that approximately 0.5%–1% of the world's population is affected by RA. It is three times more common in females than males and usually occurs between the ages of 20 and 40.^1^, ^2^ There are periods of remission and relapse during the course of the disease. Although RA primarily involves the joints, since it is a systemic disease extra-articular manifestations are also common. Eye, lung, heart, skin, kidney, larynx and nervous system are mainly affected body parts.^3^, ^4^

Extra-articular involvement manifestations in RA have been clearly demonstrated. Extra-articular disease occurs in an average of 50% of patients.^5^ When this disease is evaluated in terms of the respiratory system, although the lungs come to the fore, there are also reports of laryngeal involvement. However, it is noteworthy that there are not enough publications examining nasal functions and involvement in RA. Nasal Mucociliary Clearance (NMC) is defined as the capacity of the respiratory mucosa to remove foreign particles by preserving its ciliary activity and moisture. NMC can be determined by determining the time required for the elimination of inhaled aerosols.^6^ In this study, we tried to reveal whether there is nasal involvement by examining the NMC time and the relationship between this activity and disease severity in RA patients.

## Methods

RA patients in our hospital’s rheumatology clinic and healthy controls accepted to participate in our study voluntarily between November 2020 and February 2021 were included in this study. The diagnosis of RA was made according to the American College of Rheumatology/European League Against Rheumatism classification criteria for RA in 2010.^7^ The duration of the illness (years) and the drug usage were questioned in the patient form. Disease Activity Score-28 (DAS 28) index was used to evaluate RA disease activity. In this index, 28 joints of the patients are evaluated. The number of painful, edematous joints, Erythrocyte Sedimentation Rate (ESR) and the general health of the patient were taken into consideration. According to the scoring, DAS 28 value < 2,6 was accepted as remission, DAS value < 3.2 and ≥ 2.6 as low disease activity, DAS 28 value < 5.1 and ≥ 3.2 as moderate disease activity, DAS value > 5.1 as high disease activity.^8^ Hemogram, ESR, C Reactive Protein (CRP), Rheumatoid Factor (RF), Anti Nuclear Antibody (ANA), Anti-CCP parameters were studied from the venous blood taken from the patients. Subsequently, the patients were referred to the otolaryngology clinic. If either or both of the RF or Anti-CCP values of a RA patient were positive, that patient was considered serology positive.

Routine ear, nose and throat examination was performed in the otorhinolaryngology clinic. Patients with any of the following criteria were excluded from the study; patients with allergic rhinitis, nasal polyps or septal deviation, those who have a history of nose operation, those who have additional diseases such as diabetes mellitus, hypertension or smoking, those who had an upper respiratory tract infection in the last month. NMC time was calculated by using the saccharin test. One hour before the test, the participants were asked to stay away from eating and drinking and to clean their nose. In addition, they were allowed to rest in a quiet environment in the last half hour before the test. During the test, attention was paid to the absence of dust and breeze in the room. NMC time measurement was done at a temperature of 20–24 °C and a relative humidity of 45–60 percent. While the head of patient was 10 degree flexion position, patient was warned about not to sniff, cough, sneeze, eat or drink. After the patient was seated in an upright position, 1 mm sodium saccharin (one quarter of a saccharine tablet) was placed approximately 1 cm posterior to the medial anterior border of the inferior concha. During the application, patients were asked to maintain their posture, to breathe normally and to swallow freely. Patients were warned to avoid movements such as deep breathing, coughing, speaking and sniffing. The time between the insertion of the saccharin tablet and the time the patient had the taste of saccharine was calculated with a chronometer and recorded in minutes. An approval was obtained from the local ethics committee for this study with the number 2021/038.

### Statistical analysis

Continuous variables were expressed as mean ± standard deviation or median (minimum-maximum), categorical data as numbers and percentages. In the intergroup analysis of continuous variables, normality analyzes were performed with the Kolmogorov-Smirnov Goodness of Fit Test. In the evaluation of continuous variables between the two groups, *t-*test was used if it was compatible with normal distribution, and Mann Whitney *U* test was used if it was not. Correlation analysis was performed using the Pearson correlation test. Chi-square test was used for comparison of categorical data. Analyzes were done with IBM SPSS Package Program version 24.0 (IBM Corporation, Armonk, NY, USA). Statistical significance level was taken as *p* < 0.05.

## Results

A total of 137 patients were included in this study, including 87 patients with RA and 50 healthy controls. There was no significant difference in terms of age and gender between the RA and the control groups. While the mean NMC of the RA group was 9.51 ± 3.54 min, the mean NMC of the control group was 8.69 ± 2.85 min, and there was no significant difference in NMC between the two groups (*p* > 0.05) (Table 1). ESR and CRP values were significantly higher in the RA group compared to the control group (*p* < 0.05). The mean duration of illness was 8.17 ± 8.06 years, while the mean DAS 28 was 3.23 ± 1.10 in the RA group (Table 1).

**Table 1:** Comparison of some socio-demographic variables and some blood parameters according to the groups.

Parameter	RA Group (n = 87)	Control Group (n = 50)	*p*
Age (year) (mean ± SD)	49.00 ± 12.16	46.74 ± 7.30	0.234^a^
Gender, n (%)			
Female	10 (11.5)	7 (14.0)	0.668^c^
Male	77 (88.5)	43 (86.0)	
NMC (second) (mean ± SD)	9.51 ± 3.54	8.69 ± 2.85	0.265^b^
Hb (g/L) (mean ± SD)	12.93 ± 1.39	13.36 ± 1.26	0.074^a^
ESR (mm) (mean ± SD)	29.05 ± 19.69	8.46 ± 4.58	< 0.001^a^
CRP(mg/L) (mean ± SD)	9.87 ± 14.05	3.09 ± 1.41	< 0.001^b^
Creatinin (mg/dL) (mean ± SD)	0.67 ± 0.16	0.77 ± 0.09	< 0.001^a^
ALT (U/L) (mean ± SD)	18.78 ± 13.30	19.38 ± 8.53	0.180^b^
Duration of disease (year) (mean ± SD)	8.17 ± 8.06	–	–
DAS 28 (mean ± SD)	3.23 ± 1.10	–	–

NMC, Nasal Mucociliary Clearance; Hb, Hemoglobin; ESR, Erythrocyte Sedimentation Rate; CRP, C Reactive Protein; ALT, Alanine Aminotransferase; DAS, Disease Activity Score.

a *t*-test.

b Chi-square test.

c Mann Whitney *U* test.

When the RA group was divided into group according to being positive and negative of ANA, RF, Sjogren’s syndrome, Anti-CCP and serology; It was found that NMC averages were higher in patients with Sjogren’s syndrome and those with RF and serology positivity compared to those with negativity, but this difference was not statistically significant (*p* > 0.05). When patients were evaluated in terms of Anti-CCP, it was found that the mean NMC of the RA patients with Anti-CCP positive was significantly higher than the negative ones (*p* = 0.024). DAS 28 averages did not differ in terms of these parameters (Table 2).

**Table 2:** Comparison of NMC and DAS 28 averages in the RA group according to ANA, RF, Sjogren's syndrome, Anti-CCP and serology positivity.

Parameter	NMC (mean ± SD)	*p*	DAS 28 (mean ± SD)	*p*
ANA		0.989^a^		0.496^b^
Positive (n = 27)	9.37 ± 3.24		3.11 ± 1.11	
Negative (n = 60)	9.57 ± 3.68		3.29 ± 1.10	
RF		0.835^b^		0.326^b^
Positive (n = 53)	9.57 ± 3.72		3.14 ± 1.03	
Negative (n = 34)	9.41 ± 3.28		3.38 ± 1.20	
Sjogren syndrome		0.472^a^		0.236^a^
Yes (n = 7)	10.35 ± 4.26		2.75 ± 0.71	
No (n = 80)	9.43 ± 3.49		3.28 ± 1.12	
Anti-CCP		0.024^b^		0.651^b^
Positive (n = 56)	10.14 ± 3.87		3.27 ± 1.09	
Negative (n = 31)	8.37 ± 2.50		3.16 ± 1.13	
Serology		0.214^b^		0.597^b^
Positive (n = 69)	9.75 ± 3.77		3.20 ± 1.07	
Negative (n = 18)	8.58 ± 2.29		3.36 ± 1.25	

NMC, Nasal Mucociliary Clearance; DAS, Disease Activity Score; RF, Rheumatoid Factor; ANA, Anti Nuclear Antibody; Anti-CCP, Anti Cyclic Citrulled Peptide.

a Mann Whitney *U* test.

b *t*-test.

When the correlation between NMC and disease parameters was examined, it was found that there was no correlation between NMC and disease duration, ESR, CRP and DAS 28 (*p* > 0.05) (Table 3). A positive, low-level statistically significant correlation was found between disease duration and DAS 28 averages in RA patients (*p* = 0.043, r = 0.217).

**Table 3:** Correlation between disease duration and ESR, CRP, and DAS 28 in the RA group.

		Duration of disease	ESR	CRP	DAS28
**ESR**	**r (correlation coefficient)**	−0.027			
	**p**	0.804			
	**N**	87			
**CRP**	**r (correlation coefficient)**	0.074	0.545^a^		
	**p**	0.496	0.000		
	**N**	87	87		
**DAS28**	**r (correlation coefficient)**	0.217^b^	0.219^b^	0.432^a^	
	**p**	0.043	0.042	<0.001	
	**N**	87	87	87	
**NMC**	**r (correlation coefficient)**	−0.076	0.021	−0.011	−0.087
	**p**	0.483	0.847	0.919	0.425
	**N**	87	87	87	87

NMC, Nasal Mucociliary Clearance; ESR, Erythrocyte Sedimentation Rate, CRP, C Reactive Protein; DAS, Disease Activity Score.

a Correlation is significant at the 0.01 level (2-tailed).

b Correlation is significant at the 0.05 level (2-tailed).

## Discussion

Since many rheumatic diseases have systemic involvement, the possible effects of these diseases on other systems of the body have always been the subject of researchers. The effect of rheumatological diseases on the area of otorhinolaryngology has recently attracted the attention of clinicians. There are similar studies in RA, albeit limited in number. Kırgezen et al.^4^ reported that RA should be considered in the differential diagnosis of patients presenting with vocal and laryngological problems. In this study, it was stated that the patients had more objective findings and subjective complaints in the active phase compared to the remission phase. In a review, it was stated that RA patients have a higher risk of hearing impairment compared to healthy controls and although the risk factors are multifactorial, its mechanism is not clearly understood.^9^ It is seen that there are a limited number of publications on nasal involvement and functions in RA. In a case report, a complicated case of nasal septal perforation related to drug use in a 44-year-old female RA patient was reported.^10^ The first and only study examining mucociliary clearance changes in patients with RA is made by Shelja et al. In this study of patients with a disease duration of at least 5 years or more, it was demonstrated that mucociliary clearance was abnormal in RA patients. The authors claimed that disruption in mucociliary clearance may occur due to qualitative and quantitative changes in respiratory secretions.^11^ However, the primary limitation of this study is the small number of patients. The number of patients in our study was quite sufficient, but the least illness duration of the our patients was one year. In our study, NMC averages of both the RA and the control groups were within normal limits, and there was no significant difference between the groups in terms of NMC averages. However, the average DAS 28 of RA patients was 3.23, and in this respect, it can be said that our study patients consisted of patients with low disease activity. The lack of a significant difference in terms of NMC times between the RA and the control groups in this study can be explained by this reason.

The prevalence of airway disease is very high in RA, it can occur in 39%–60% of patients on average. Any part of the airway may be involved, including the large airways (upper and lower) and distal small airways. The lung is usually the most frequently involved extra-articular disease site. The most common manifestations are bronchiectasis, bronchiolitis, airway hyperreactivity and cricoarytenoid arthritis. RA patients with extra-articular symptoms have increased mortality compared to patients without, and the main causes of this situation are cardiovascular disease, infections and lung disease.^12^ The prevalence of laryngeal involvement in RA varies between 13% and 75%.^13^ It has also been reported that laryngeal symptoms and signs may be more common in patients with longer disease duration.^4^ However, although the involvement of the respiratory tract in RA has been clearly demonstrated, we still do not have enough information about nasal involvement. In this study, while there was a correlation between disease duration and DAS 28 average, no correlation was found between NMC time and DAS 28. In addition, it was found that there was no correlation between NMC and disease duration, ESR and CRP values. However, as stated above, our patients consisted of patients with mild and moderate disease activity. Therefore, we think that making this correlation with patients with moderate and severe disease activity will yield more accurate results.

RA is a chronic, progressive inflammatory condition and is associated with increased mortality due to the cardiovascular diseases and infections. Common infections in RA patients may be due to the disease itself, extra-articular manifestations, or disease-modifying drugs.^14^, ^15^ In a study involving 609 RA patients, it was reported that RA patients had twice the infection rate compared to controls.^16^ In addition, it has been claimed that environmental respiratory viral diseases are associated with an increased number of RA cases, especially in elderly and female patients, and that respiratory viral infections may be an environmental risk factor for the development of RA.^17^ In the hypothesis that RA may be of mucosal origin, it is claimed that the disease originates from one or more mucosa. In this hypothesis, it has been proposed that the most important early event in the preclinical development of RA is not loss of tolerance to self-antigens, but loss of mucosal barrier function and systemic dissemination of an IgG anti-citrullinated protein antibody response.^18^ Therefore, in the light of these data, it can be clearly stated that upper respiratory tract infections, including the nasal mucosa, may appear both as a cause and as a result in the etiopathogenesis of RA. For this reason, we aimed to reveal the nasal physiopathological changes in RA patients in this study. NMC is an important mechanism that removes foreign particles and pathogens and keeps the mucosa moist and fresh.^19^ NMC is the primary defense mechanism of the upper and lower airways and any disruption of this process leads to nasal, paranasal and airway infections.^20^ In this study, it was found that there was no significant difference in terms of NMC time between RA patients and healthy controls. However, NMC of anti-CCP positive RA patients were significantly higher than those with Anti-CCP negative. The presence or absence of this antibody allows RA to be distinguished from other rheumatic diseases. In addition, the titer of anti-CCP can be used to determine the prognosis of the disease and predict the treatment outcome.^21^ Tein Tsai Cheng et al.^22^ reported that the probability of a 10-year and higher hip fracture in anti-CCP positive RA patients was higher than in anti-CCP negative patients. In our study, it was found that there was a relationship between anti-CCP positivity and increased NMC.

The main limitation of this study is that our patient group was not fully homogeneous in terms of drug use. However, since many drugs can be used in RA patients, we think that it will be difficult to make a homogeneous distribution in terms of drug use in these patients. It would be a more accurate method to calculate the NMC time when patients were not taking medication, but we could not do this method because it would be difficult to create this patient population.

## Conclusion

In this study, there was no significant difference in NMC time between the RA patients and the control group, and the NMC time of both groups was within normal limits but NMC was higher in patients with anti-CCP positive RA patients. We think that performing this study with patients with moderate and severe disease severity may contribute to the literature.

## Compliance with ethical standards

This research did not receive any specific grant from funding agencies in the public, commercial, or not-for-profit sectors.

Permission was obtained from the ethics committee of Karatay University for this study (nº 2021-038). We have worked in accordance with the Helsinki Declaration and subsequent amendments.

Written informed consent forms were received from all participants.

There is no author identifying information anywhere in the blinded manuscript.

## Conflicts of interest

The authors declare no conflicts of interest.

## CRediT authorship contribution statement

**Hilal Yücel:** Conceptualization, Data curation, Formal analysis, Funding acquisition, Methodology, Writing – original draft. **Serpil Ergülü Eşmen:** Conceptualization, Data curation, Formal analysis, Funding acquisition, Methodology, Writing – review & editing.
